# Taxing energy to tackle greenhouse gases: evaluating the role of financial risk in high-income economies

**DOI:** 10.1007/s11356-023-30310-4

**Published:** 2023-11-08

**Authors:** Taimoor Hassan

**Affiliations:** Faculty of Economics and Sociology, Department of Macroeconomics, University of Lodz, Lodz, Poland

**Keywords:** Greenhouse gases, Energy tax: Digitalization, Consumption of renewable energy, Financial risk

## Abstract

Energy, a basic input to the economic system, plays a pivotal function in development; at the same time, it raises concerns and hurdles to global economies as a result of negative externalities associated with its usage. Economies set various measures to limit these negative externalities and encourage citizens toward renewable energy utilization. Considering a panel of high-income economies over the period of 1990–2020, we empirically examine whether energy-related tax policies (ENT) are helpful to tackle the issue of energy-related greenhouse gas emissions (ENGHGs). Furthermore, we also investigate the role of digitalization (DIG) and financial risk (FINR) for its possible impact on ENGHGs. The advanced econometric techniques include diagnostic tests, Method of Moment Quantile Regression (MMQR), for robustness validation quantile regression, and finally Dumitrescu and Hurlin panel causality check. The findings reveal that ENT policies of selected economies are not helpful to limit ENGHGs in 25th and 50th quantiles effectively. Nevertheless, due to the progressive rise in ENT in the 75th and 90th quantiles, ENT significantly helps to smoothen the path towards a sustainable future. Furthermore, GDP increases, while improvement in FINR decreases ENGHGs. As the selected economies are developed and high-income, it is suggested that a progressive rise in ENT may further limit the issue of ENGHGs.

## Introduction

**Fig. 1:**
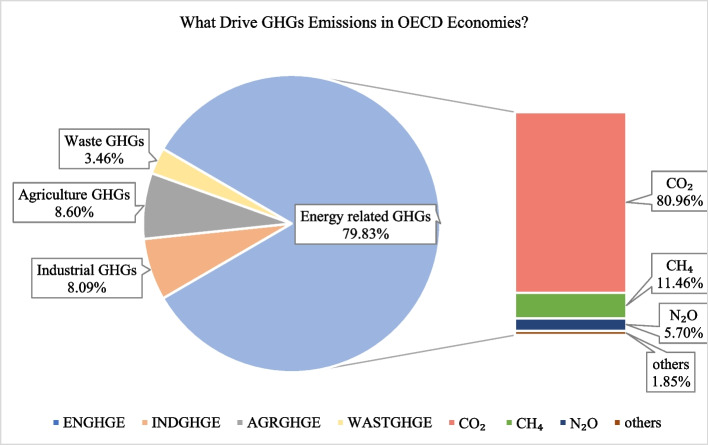
Drivers of GHG emissions in the selected OECD member states based on the data gleaned from UNFCCC (1990-2019)(“United Nations Framework Convention on Climate Change (https://di.unfccc.int/time_series)”)

Since the early stage of industrialization in the seventeenth century, anthropogenic activities have summed up an extensive quantity of greenhouse gases (GHGs) in the climate due to ramping up dependency on fossil fuels, replacing forest areas with unsustainable communities in result of rapid pace of urbanization. The heterogeneous share of GHGs emissions of each country across the globe explains several factors such as economic structure (including how efficient are resources utilized), environment-related policies, and socio-economic conditions— such as level of education, income level, population, land, and climate conditions, which effectively determine the GHG emissions of a country. The primary cause of changing climate is global in scale, and hence, monitoring and examining emissions globally will enable a stronger insight of each nation’s struggle to combat climate change while bringing up the matter of hazardous emissions, which will instruct economies to reshape their environment-related laws with the aim of achieving climate-resilient societies in the near future. The collective warming effect from GHGs attributed to the planet’s atmosphere by human involvement has surged by 45% from 1990 to 2019. Energy generation and consumption is the leading source of GHG emissions globally, responsible for 79% of all GHGs emissions in 2015 (IPCC 2014). From the data description in Fig. 1, GHGs arising from the energy sector are the leading source of GHG emissions, where CO_2_ dominates energy-related greenhouse gases emissions (ENGHGs) with a huge share of 80.96% in the OECD member states.

**Fig. 2:**
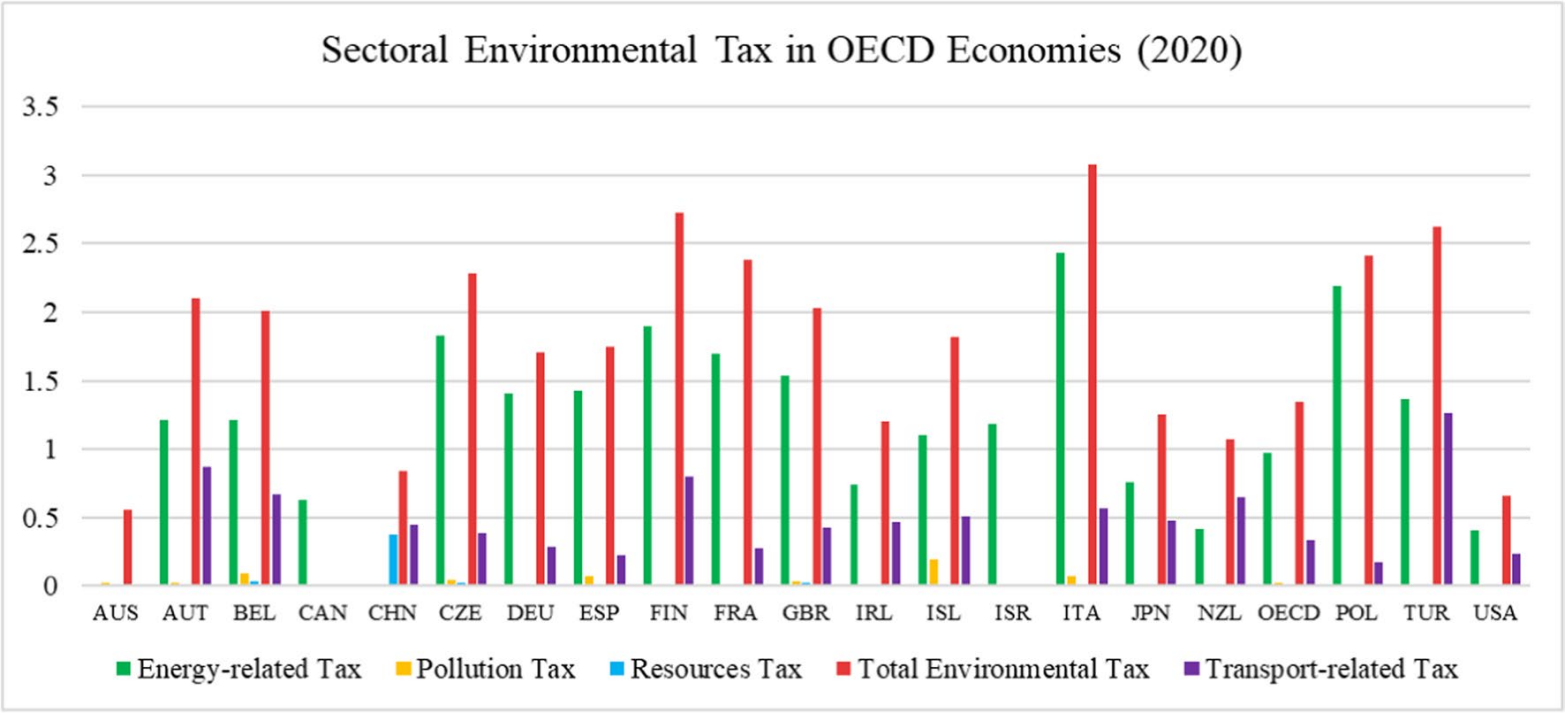
Sectoral environmental tax in OECD economies (2020)

**Fig. 3:**
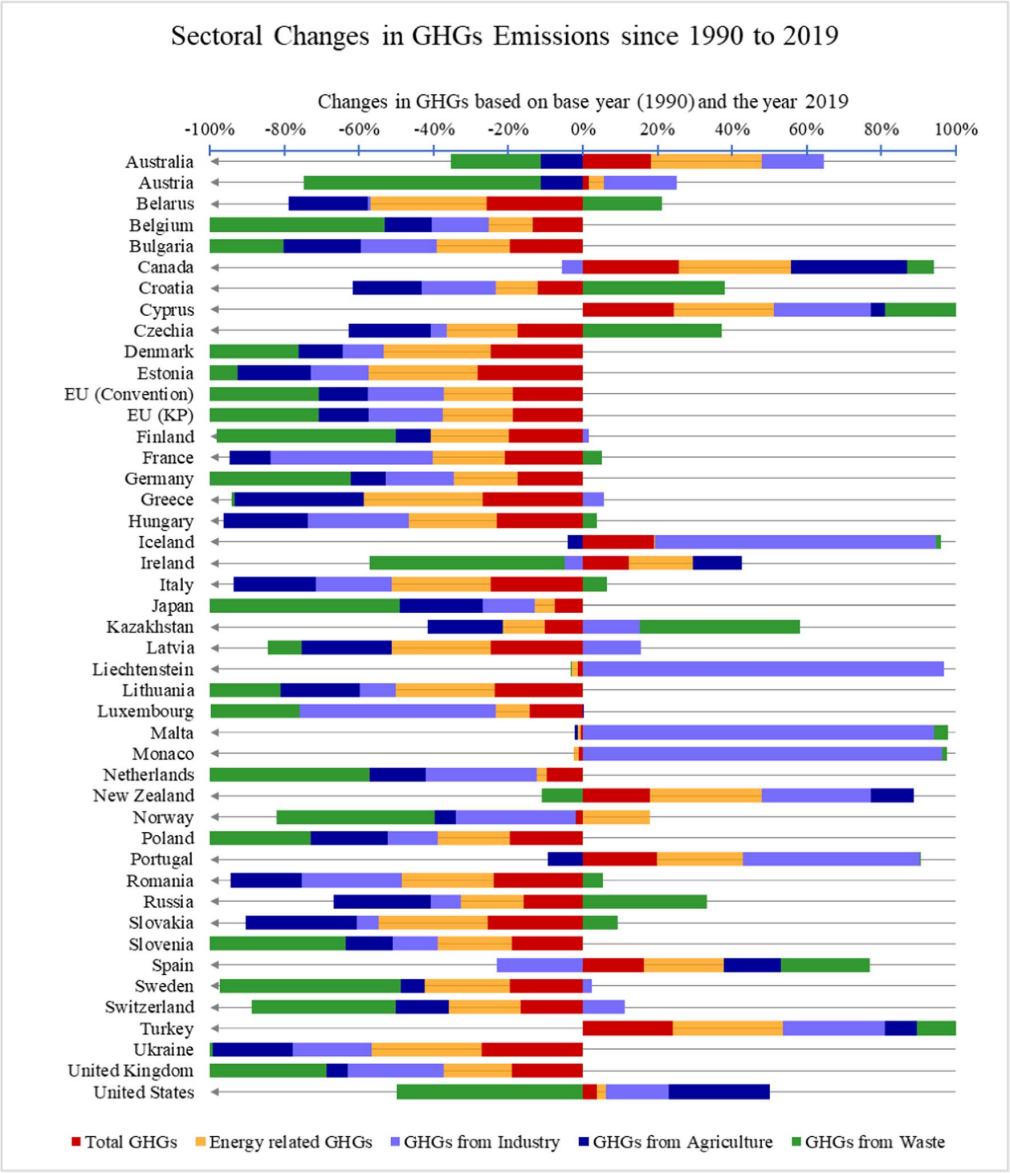
Sectoral changes in GHG emissions (1990–2019) based on the data gleaned from UNFCCC

The data from UNFCCC (1990-2019), visualized in Figs. 2 and 3, highlights the changes in GHG emissions based on the four different sectors. It is clear from Fig. 3 that most of the OECD states have effectively tackled GHG emission sourcing from energy, industry, agriculture, and waste sectors since 1990 to 2019. The largest increase of 130% in the total GHGs emissions was recorded for Turkey in 2019 compared to the base year 1990, whereas the largest decrease of 64% in the total GHG emissions was recorded for Ukraine. The largest rise in ENGHGs emissions of 161% was recorded as the highest increase in ENGHG emissions among all countries in the year 2019 since 1990. At the same time, Ukraine has shown great achievement by curbing 69% of its ENGHGs emissions in 2019.

In the last decade, a growing number of governments throughout the world have acknowledged climate change–related concerns. Extreme weather phenomena such as soil degradation, wildfire, flooding, typhoons, rising sea level, reef bleaching, and heat waves are also a matter of concern. Furthermore, several European Union (EU) member nations have recently enacted various rigorous environmental policies, including market instruments such as tax and pricing systems, as well as non-market instruments such as the execution of environmental rules and standards. Environmental laws may have a substantial influence on nations all over the globe in terms of comparative advantage. Particularly, industrialized economies shift their filthy businesses to economies with low environmental standards. Hence, there is plenty of evidence to incorporate environmental regulations or tax as a possible explanatory variable while modeling for environmental deterioration (Chien et al. 2021; Hassan et al. 2022a; Li et al. 2021b).

A number of studies have been performed on the connection between environmental pollution and its drivers (Safi et al. 2021b; Wahab et al. 2022; Ulucak and Khan, 2020; Cai et al. 2018; Hassan et al. 2023; Wahab, 2021; Dogan and Seker 2016; Hassan et al. 2022a; Khan et al. 2021; Ozturk and Acaravci 2013). Recently, a number of academics have concentrated on the effect of financial risk (FINR) on CO_2_ emissions, although they have yet to reach a consensus. For example, Zhang and Chiu (2020) examined the nonlinear influence of FINR on carbon emissions by employing a panel smooth transition regression model and discovered a negative connection between FINR and emissions using data from 1985 to 2014. Furthermore, Shahbaz et al. (2017), Zaidi et al. (2019), and Abbasi and Riaz (2016) found a negative FINR and emission connectivity, which is consistent with Zhang and Chiu (2020). Besides, several researches have confirmed that FINR deteriorates the quality of the environment. For example, Zhao et al. (2021) revealed that FINR activates emissions in China, which is further supported by Shahbaz et al. (2013) whereas, Abbasi and Riaz (2016) revealed that FINR had no significant influence on emission levels.

Multiple studies have been conducted over the last two decades to investigate the causes of environmental degradation and to develop ecologically friendly solutions toward a sustainable future. In this context, choosing the most appropriate proxy of environmental pollution is crucial as there are several available proxies for estimating environmental pollution, for instance, carbon dioxide emissions, ecological footprint, trade-adjusted carbon emission, and emissions of greenhouse gases. Previously, researchers highlighted various factors affecting carbon emissions (Dogan and Seker 2016; Hassan et al. 2022a; Khan et al. 2021; Ozturk and Acaravci 2013; Zaidi et al. 2019; Zhang and Chiu 2020; Zhang 2011). While other researchers such as Ahmed et al. (2019), Hassan et al. (2019), Hassan et al. (2022b), Kirikkaleli et al. (2021), Ulucak and Khan et al. (2020) employed ecological footprint as a proxy element for environmental pollution. Nevertheless, few researches have looked at ENGHGs as a cause of environmental deterioration. For instance, an early study of Tian et al. (2013) examines ENGHGs of the Chinese iron and steel industry in China. In the context of ENGHGs, most of the available studies focus on China, while there are limited studies that consider panel data for the empirical findings of ENGHGs. For instance, a recent study published in Nature Energy by Nielsen et al. (2021) examined the impact of people of high socioeconomic status on ENGHGs in Europe and North America. The present study seeks to address the primary research issue of environmental externalities. The research topic is connected to concerns of progressive increase in ENGHGs. The key goal of this article is to empirically explore the influence of key important determinants of ENGHGs. Expanding energy security and decarbonization concerns have recently prompted numerous countries to adopt environmental taxes. More particular, it is crucial for wealthy and industrialized countries that environmental policies (taxes, technology, and energy) genuinely contribute toward cleaner production and a sustainable future environment.

The remaining of the article is structured as “Literature review,” consisting of previous related studies, methodology and empirical work are reported in “Methodology and empirical work,” econometric findings of this study are discussed in “Econometric findings and discussions,” whereas conclusions and policy recommendations are mentioned in the last section.

## Literature review

Extensive research has been conducted to evaluate the association between environmental degradation and economic development. However, in recent years, adverse environmental effects have been the core avenue of economic growth, considering that GHG emissions create an irreversible distraction to economic progress (Nations 2015; Ulgiati et al. 1995). Meanwhile, carbon dioxide emissions generate a vast amount of GHG emissions, which substantially contribute to accelerating worldwide temperature and are linked with the instability of climate change (Tiwari et al. 2013; Wen et al. 2011). The following researchers’ hypotheses are objecting against the traditional economic theory, which accounts for a trade-off between quality of environmental and economic development (Meadows et al. 1972). Nevertheless, published studies proposed the nexuses between environmental mitigation variations and economic growth, environmental taxes, FINR, and DIG in the selected high-income countries. The literature suggests that the association between the variables is positive when economies surpass a specific income level (Grossman and Krueger 1991; Panayotou 1993).

Hypothesis I. GDP positively affects energy-related GHG emissions.

### The link between GDP and GHG resulting from energy

Many scholars in the current literature address the connection between economic development and GHGs. Researchers believe that economic expansion has an inverse association with climate change. Bengochea-Morancho et al. (2001) revealed that due to a significant disparity in economic development among many European economies, each country should take into account specific industrial structures and economic activities of overcoming the adverse impact of environmental pollution. Chebbi et al. (2011) examined the relationship between carbon emissions and trade openness and found an adverse effect of CO_2_ emissions on economic development in the long term. Mehrara (2007) studied the casual association between GDP and energy usage in major oil-exporting nations. The results of his investigation demonstrate a one-way causal relationship between economic growth and energy use. Furthermore, he advocates for energy price restructuring in the following nations while maintaining economic growth and improving quality of environment. The study developed by Yoo and Jung (2005) confirmed a one-way casual linkage between nuclear energy utilization and economic development in both the short and long run for Korea. Using the Granger causality test provided by Toda and Yamamoto (1995), Heo et al. (2011), and Wolde-Rufael (2010) found a one-way causal link between nuclear energy and economic progress. Additionally, it appears that there is a two-way causative relationship between economic growth and the use of nuclear energy in the short term, whereas in the long run, there is only a one-way causal relationship between nuclear energy consumption and economic growth (Apergis and Payne 2010).

Hypothesis II. Environmental taxes are negatively linked with ENGHGs.

### The nexus between environmental taxes and ENGHGs

The current literature has greatly focused on the various linkages that may happen between energy consumption, environmental proxies, economic development, and environmental tax that may have an effect on the level of pollution. The findings of Aydin and Esen (2018) confirm an asymmetrical association and discover the exceeding level of threshold, the outcome of taxes on carbon emission alterations from statistically insignificant positive association to substantial negative in 15 EU partner countries over 1995–2013. The latest study proposed by Mardones and Cabello (2019) found that environmental tax scenario for SO_2_, CO_2_, and PM emissions would fall by 49%, 11%, and 45% in Chile. Similarly, Peng et al. (2019) reveal that taxes and energy can be an important factor for conservation and energy-saving purposes. In addition, Miller and Vela (2013) suggest that countries with high taxes reveal a remarkable decrease in the production of non-renewable energy and CO_2_ emissions. Furthermore, the findings of Morley (2012) confirmed an inverse link between environmental taxes and pollution in the EU; however, their analysis found no relationship between energy consumption and taxation.

Hypothesis III. Higher financial risk increases, while lower financial risk decreases ENGHGs.

### The impact of FINR on ENGHGs

In the past years, many researchers have focused substantially on the significance of FINR on CO_2_ emissions. In our study, we explore the impact of FINR on ENGHGs for the high-income OECD economies. Ozturk and Acaravci (2013) revealed that long-term financial stability has an influence on Turkey’s CO_2_ emissions. The study proposed by Zhang and Chiu (2020) uses a panel for 111 economies over the period of 1985 to 2014 to examine the nonlinear impacts of different countries’ inclusive risks on carbon emissions. Based on the econometric analysis, higher FINR can significantly increase the level of CO_2_ emissions. Similarly, the study introduced by Abbasi and Riaz (2016), Zaidi et al. (2019), and Shahbaz et al. (2018) found the same outcomes. On the other hand, Zhang (2011) suggests that financial stability is a significant factor for improving CO_2_ emissions in China.

Hypothesis IV. Digitalization curb of ENGHGs.

### The effects of DIG on ENGHGs

In the DIG-environment nexus, the present literature provides contradictory findings; some researchers state a positive association between DIG and environment, while others believe that DIG helps sustain environmental quality (Vial, 2021). Believers of the first view asses that in the ages of internet development, various negative externalities arise as a byproduct affecting environmental quality (Feroz et al. 2021; Salahuddin and Alam 2016). Similarly, the enhancement of internet and technology has triggered an increase in electricity consumption demand, which ultimately leads to the exploitation and depletion of resources (Majeed and Luni 2019). Besides, some asserted that DIG smoothens the path toward sustainable development and cleaner energy transition (Feroz et al. 2021). The believers of the second view support that DIG has enhanced technological advancement in the past few years and is crucial in switching toward a circular economy (Holger Berg et al. 2020). Companies may also boost economic policies that cut ENGHGs by using AI, IoT, and other technology-based data analytics (Demartini et al. 2019). AI application is also seen as a promising strategy for addressing the challenges of distinctive, interactive, and changeable ecological circumstances (Ye et al. 2020). The environmental impact of digitization is also seen on the demand side, with customers shifting to cleaner resources and demanding more eco-friendly products as DIG spreads via numerous channels (Energiewende 2019).

In response to the growing threat of climate change and rising global average temperatures, organizations and researchers across the globe have concentrated on finding new routes to a sustainable future and reducing anthropogenic emissions of GHG. In this context, plenty of available research empirically identifies various drivers of environmental pollution. Based on the employed proxy of environmental pollution, these researchers have incorporated different proxies of environmental pollution. For instance, Ding et al. (2021), Fernández-Amador et al. (2017), Hassan et al. (2022a), Hassan et al. (2021), and Khan et al. (2020) considered trade-adjusted carbon emissions, while Ahmed et al. (2019), Alola et al. (2019), Aydin et al. (2019), Danish and Khan (2019), Hassan et al. (2022b), and Ulucak and Khan et al. (2020) employed ecological footprint of consumption. As a fundamental component of the economic system, energy is considered the crucial element in the changing climate and environmental pollution debates (Hassan et al. 2022b). However, very few studies specifically focus on the emission associated with the energy consumption or production (Wang et al. 2017; Zhang and Da 2015). Nevertheless, these studies consider energy-related carbon emissions as a dependent variable in their empirical model. Moreover, these studies are based on China’s provincial or city-level data. There is a lack of studies focused specifically on GHGs associated with the energy sector in the developed economies. Moreover, the present study also incorporates other interesting factors such as DIG and FINR for examining its possible influence on ENGHGs in a panel of high-income economies over the past 31 years.

## Methodology and empirical work

### Theoretical setup

**Fig. 4:**
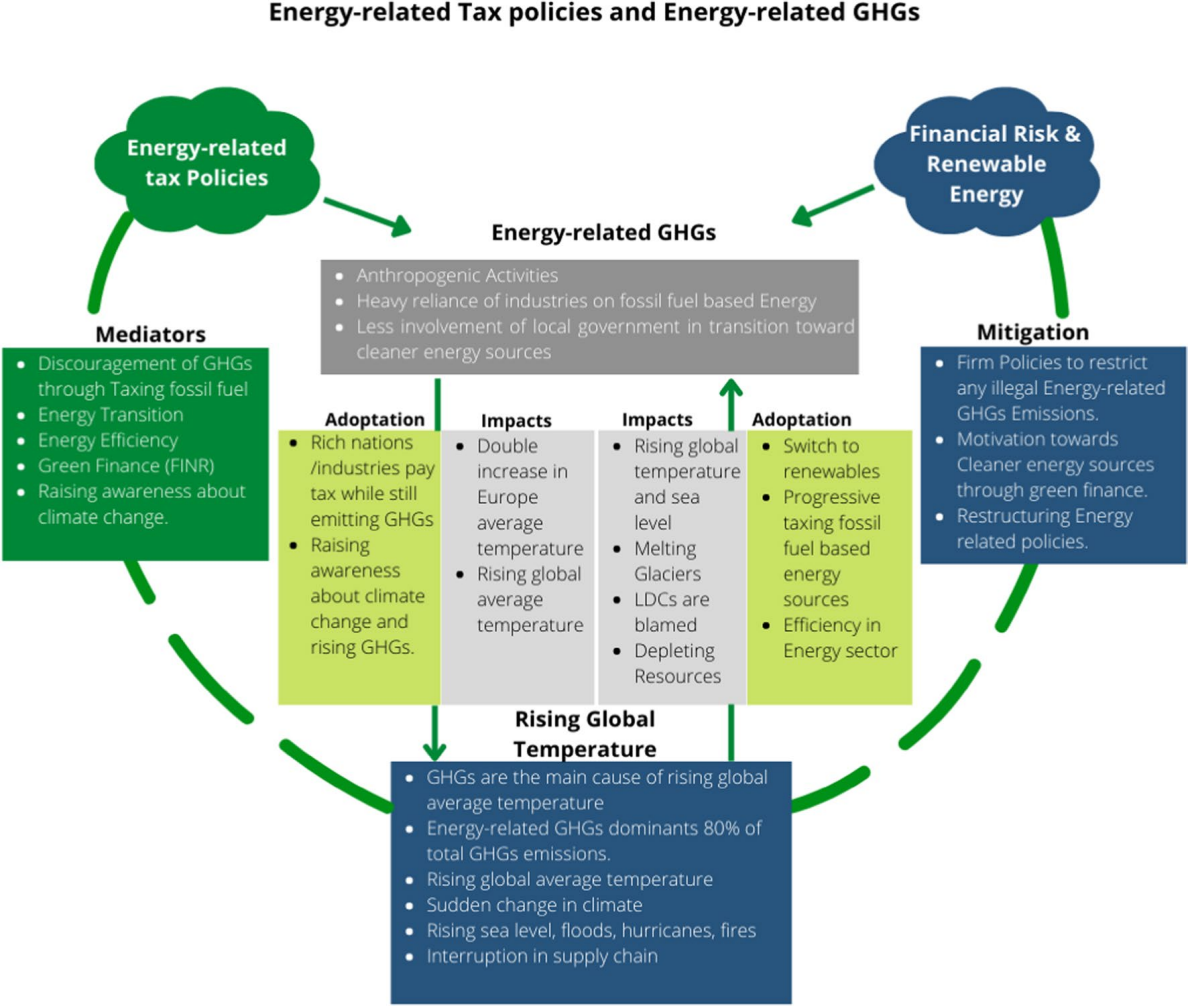
Impact mechanism and mediating channels

Global concerns over changing climate are mounting, and rising temperatures necessitate the investigation of new possibilities for an effective energy transformation and environmental protection induced by various anthropogenic activities. Developed countries are committed to reducing their environmental impact more efficiently through tougher environmental laws. However, due to high-income per capita, availability, and affordability of energy sources, it is rational that developed economies consume more energy compared to lower-income economies. In this context, it is noteworthy to report that though developed economies are moving toward environmental sustainability, in the meantime, they hold the largest share in the ENGHG emissions. Therefore, the present study is based on a panel of selected high-income OECD economies to explore the possible key determinants of ENGHGs (Fig. 4).

As stated by EKC hypothesis, as incomes improve, the quality of the environment typically degrades. Nevertheless, after a certain level of income is attained, a rise in income level is no longer linked with environmental degradation but instead improves environmental quality. This statement has previously been justified in various literary studies for some regions (Balsalobre-Lorente et al. 2017; Bilgili et al. 2016; Cole 2004; Destek and Sarkodie 2019; Ișik et al. 2020; Khan and Bin 2020). For instance, Destek (Destek and Sarkodie 2019) confirmed the validity of an inverted U-shaped EKC for newly industrialized nations. As countries expand, it creates job opportunities, maximizing production capabilities, and booming economic activities, generating rise in income (Hassan et al. 2022b; Hassan et al. 2021). In this context, the rise in income level results in enhancing standard of living, gaining access to various other facilities previously restricted due to lower income level. As a result, rising income levels activate higher consumption of energy sources, causing an increase in emissions (Hassan et al. 2022a; Khan et al. 2020). After reaching a particular amount of income, people adopt to become more sensitive to environmental externalities and prefer less low-carbon activities and products. However, literary studies reported that validity of EKC hypothesis does not prevail for all nations homogeneously and there might be other factors linked to environmental pollution (Al-Mulali et al. 2016). Taking into consideration the first stage of EKC, we expect GDP as detrimental for environmental quality.

Besides income level, it is generally believed that imposing stringent environmental measures restrict human-induced environmental externalities resulting in emissions reduction (Chien et al. 2021; Ghazouani et al. 2021; Li et al. 2021a). This statement is also supported by well-known Porter hypothesis (Porter and Van der Linde 1995). The world’s energy consumption increased significantly in 2018, as did greenhouse gas emissions connected to energy, which hit a new all-time maximum. This is concerning since fulfilling the Paris Agreement’s targets would need significant reductions in emissions. The OECD report on *Taxing Energy Use 2019* states that tax structures in the region are not well linked with the emission profiles of energy sources; despite its negative climate and air pollution implications, coal is taxed at very low or nil rates (OECD 2019). Moreover, such instant energy sources are comparatively cheaper but pose a serious threat to global environmental quality. In this context, we theorized that the energy-related tax policies in the selected high-income economies since 1990–2010 may not effectively restrict ENGHGs.

According to the *World Economic Forum Annual Meeting 2022* (Manju George and Holst 2022), the consequences of climate change are becoming more extreme by the day, while promises for 2030 are only expected to lower emissions by 7.5%. To keep the Paris Agreement targets on pace, we need a 55% cut by 2030. To close this gap, high-emitting industries must be rewired around efficiency, circularity, and sustainability. Digital technologies can aid this shift. Energy, materials, and mobility are the three most polluting sectors, accounting for 34%, 21%, and 19% of total 2020 emissions, respectively. They also represent industries with the greatest potential to minimize emissions from digital technology. Therefore, we expect that digital economy could enhance efficiency in energy sector by minimizing the intensity of ENGHGs.

FINR assesses a country’s ability to fund state agencies such as governmental, commercial, and trade debt commitments. To sum it up, a lower FINR indicates that an economy is more productive and capable of returning its loan. Moreover, because of lower FINR, nations with stable currency rates attract international investments. Financial volatility and technological developments, according to Safi et al. (2021a), improve environmental quality. This implies that economic operations in unstable countries are inefficient because of inadequate production capabilities, a lack of finance for government activities, a large overseas debt, and an uncertain exchange rate, among other issues. According to a research by Zhang (2011), China’s increased carbon emissions are significantly attributed to lower FINR (financial growth). Therefore, we anticipate that the lower FINR of an economy has an inverse linkage with ENGHGs in the selected high-income economies. Hence, we expect that improvement in FINR may tackle the issue of ENGHGs in our investigation.

### Model specification

To explore the empirical association of energy-related tax policies, GDP, FINR, ENT, and DIG on ENGHGs, this study proposed the following model:

1 $$ENGHGsi,t={\zeta}_1 GDP+{\zeta}_2 FINR+{\zeta}_3 DIG+{\zeta}_4 ENT+{\epsilon}_{it}$$

where ENGHGs represent emissions of energy-related greenhouse gases; GDP stands for gross domestic product, FINR is denoted by FINR, DIG is mentioned by DIG, and ENT represents energy-related environmental tax. On the other hand, *ζ*’s are the coefficients or degree of magnitude, and *ϵ* is the error term. Prior to the basic empirical estimation, the data is converted to logarithmic specification. The log transformation simplifies the interpretation of coefficients and estimates while maintaining a constant variance.

### Data description

**Fig. 5:**
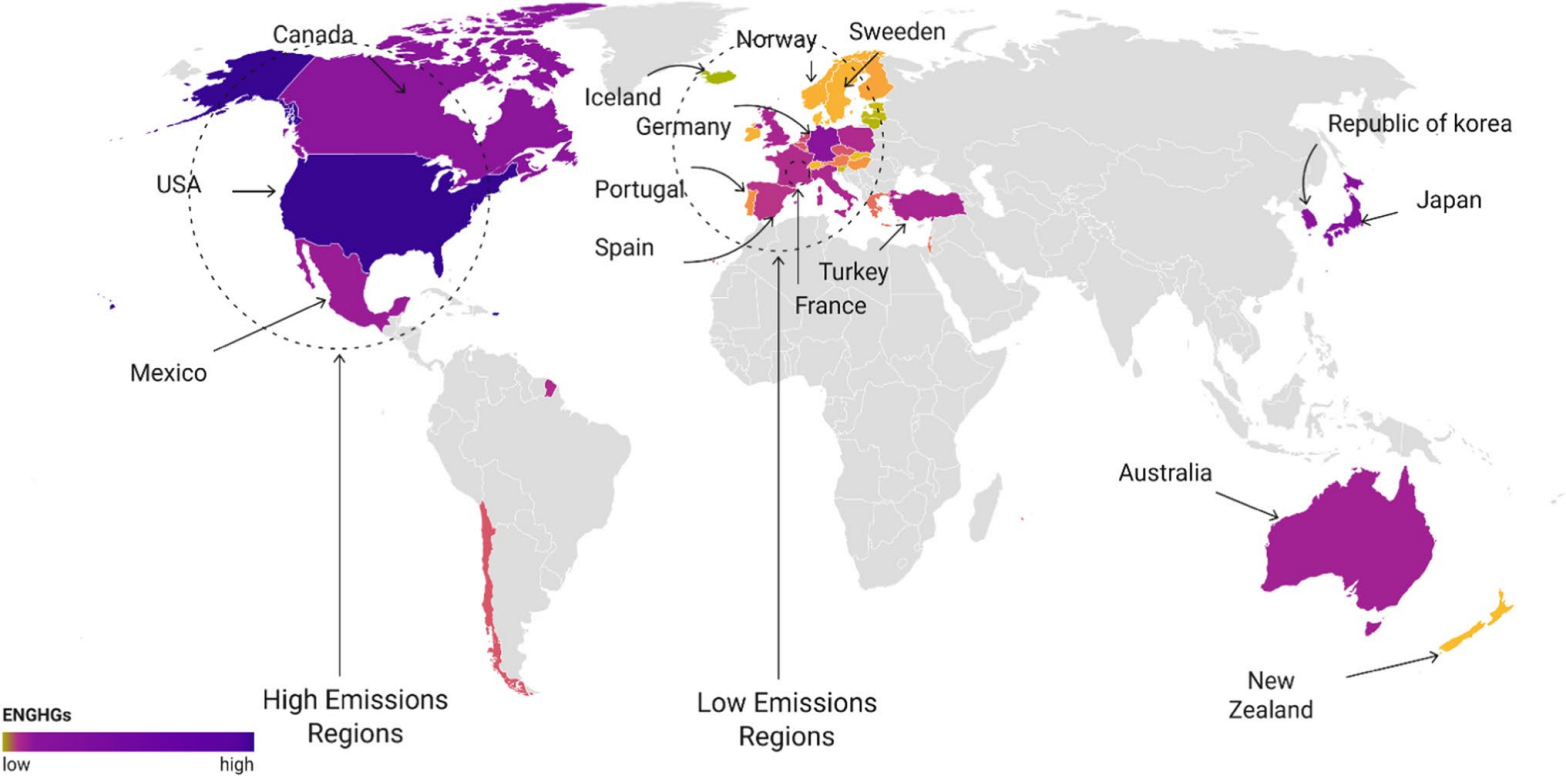
ENGHGs in OECD economies

The current study empirically investigates whether any fluctuation in GDP, FINR, DIG, and ENT has repercussions in the ENGHGs of selected high-income economies over the period of 1990–2022. Table 1 shows the variables’ notations, definitions, measurement units, data sources, and predicted connections. Energy-related greenhouse gas emissions (ENGHGs) measured in tons of CO_2_ equivalent in thousands are the target variable in the study model. The adjusted tax list is compiled at the national level across a variety of environment-related tax categories. Energy items (fossil fuels and electricity), particularly those used in transportation (petrol and diesel), are subject to the energy-related tax. The economies under investigation include “France, Germany, Israel, Austria, Australia, Slovak-Republic, Switzerland, Turkey, United Kingdom, United States Canada, Chile, Czech Rica, Belgium, Denmark, Estonia, Greece, Hungary, Iceland, Ireland, Finland, Italy, Japan, South Korea, Latvia, Mexico, Netherland, New Zealand, Lithuania, Luxemburg, Norway, Poland, Portugal, Slovenia, Spain, and Sweden” (Fig. 5).

**Table 1:** Data explanation

Variables	Notation and units	Source	Expected sign
ENGHGs	ENGHGs (tons of CO_2_ equivalent, thousands)	http://www.stats.oecd.org	--
GDP	GDP (constant 2015 US$)	World Bank	Positive
FINR	FINR Index	ICRG	Negative
DIG	Individuals using the Internet (% of population)	World Bank	Negative
ENT	Energy related tax revenue, % of GDP	http://www.stats.oecd.org	Negative

### Diagnostic tests

Due to greater dependency and connectivity among regions, economies are more susceptible to external shocks and global events. While handling panel data for econometric analyses, cross-sectional dependency (CD) and slope heterogeneity (SH) problems commonly occur. Overlooking the CD and SH tests may lead to incorrect econometric procedures and erroneous and biased regression findings. In this context, prior to the econometric analyses, we applied Pesaran's (2004) test for observing CD in the panel. The equation is based on the assumption of a zero mean constant and variance. In the following equation, the term “$\hat{\tau}$*ik*” represents pairwise correlation. The test equation is as follows;

1 $${CD}_{pesaran(2004),i}=\sqrt{\frac{2\textrm{T}}{N\left(N-1\right)}}\left(\sum_{i=1}^{N-1}\sum_{k=i+k}^N\hat{\tau} ik\right)$$

The slope heterogeneity test (Pesaran and Yamagata 2008) is employed in this study. The technique is beneficial for testing and informing the researcher about the presence or absence of SH. Based on the findings of this test, a more rigorous unit-root and cointegration test would be used. Equation 2 A may be used to calculate the Delta tilde value:

2A $${\overset{\sim }{\Delta }}_{Slope- Heterogeneity}={(N)}^{\frac{1}{2}}{(2k)}^{-\frac{1}{2}}\left(\frac{1}{N}\overset{\sim }{S}-k\right)$$

2B $${\overset{\sim }{\Delta }}_{Adjusted- Slope- Heterogeneity}={(N)}^{\frac{1}{2}}{\left(\frac{2k\Big(T-k-1}{T+1}\right)}^{-\frac{1}{2}}\left(\frac{1}{N}\overset{\sim }{S}-2k\right)$$

#### Panel unit root testing

There are various unit-root tests to assess the integration order of the variables. These tests differ in their requirements and assumptions. The primary distinction between the tests (Levin et al. 2002; Breitung 2001; Im et al. 2003) is that the former two presume homogeneity whereas the latter implies heterogeneity across cross-sections. However, these techniques cannot cope with heterogeneity and cross-section dependency. Considering CD and SH, this study used the augmented cross-sectional IPS (CIPS) test, the test is effective since it handles the CD variables and delivers consistent outcomes in SH (Pesaran 2007). The generic equation is as follows:

3A $$\Delta {Z}_{i,t}={\delta}_i+{\delta}_i{X}_{i,t-1}+{\delta}_i{\overline{Z}}_{t-1}+\sum_{l=0}^p{\delta}_{il}\Delta \overline{Z_{t-l}}+\sum_{l=1}^p{\delta}_{il}\Delta {Z}_{i,t-l}+{\varepsilon}_{it}$$

${\overline{Z}}_{t-1}$ and $\Delta \overline{Z_{t-l}}$ indicate the averages and lags described in Eq. 3A. The CDF value is generated using the equation, and the CIPS is displayed as:

3B $$\hat{CIPS}=\frac{1}{N}\sum_{i=1}^n{CDF}_i$$

#### Westerlund panel cointegration (ECM)

To confirm the cointegration of the variables, we apply the Westerlund (2007) cointegration test. This test outperforms Pedroni (2001) and Kao (1999) by considering the issue of CD and SH. The test provides *G*_*t*_ and *G*_*a*_ for group and *P*_*a*_ and *P*_*t*_ for panel statistics reported as follows:

4A $${G}_t={N}^{-1}\sum_{i=1}^N\frac{{\hat{\vartheta}}_i}{Standard\ Error\ \left({\hat{\vartheta}}_i\right)}$$

4B $${G}_a={N}^{-1}\sum_{i=1}^N\frac{T{\hat{\vartheta}}_i}{{\hat{\vartheta}}_i(1)}$$

The group statistics are analyzed for null of no cointegration and alternative as cointegration for the entire panel.

4C $${P}_t=\frac{\hat{\vartheta}}{Standard\ Error\ \left(\hat{\vartheta}\right)}$$

4D $${P}_a=T\hat{\vartheta}$$

According to the panel test statistics, at minimum, one of the cross-sections is cointegrated.

### Method of moment quantile regression

In accordance to the Jarque and Bera (1980) test in the descriptive statistics table, it is clear that the data under examination in this study is not normally distributed as we reject the null of the Jarque and Bera test at a high level of significance. As a result, we apply Machado and Silva's (2019) method of moment quantile regression (MMQR). The typical quantile regression methodology does not deal with the problem of overlooked heterogeneity. On the other hand, MMQR is an effective approach for investigating the conditional heterogeneous covariance influence of GDP, FINR, DIG, and ENT on ENGHGs. According to Machado and Silva (2019) and several others, the regression is as follows:

5 $${Y}_{\rho}\left({~}^{\tau }\!\left/ \!{~}_{{X}_{i,t}}\right.\right)={\sigma}_1+{X_{i,t}}^{\prime}\zeta +\left({\alpha}_i+{Z_{i,t}}^{\prime}\gamma \right){U}_{i,t}$$

“*Y*_*ρ*_” represents the dependent variable, whereas “*X*_*i*, *t*_” represents all regressors. The scale parameter “(*α*_*i*_ + *Z*_*i*, *t*_^′^*γ*)*U*_*i*, *t*_” denotes the quantile fixed effect “*i*” over the cross section units “*t*”. Moreover, to confirm the MMQR findings, we employ a simple Quantile regression approach.

## Econometric findings and discussions

The outcomes of the econometric techniques previously mentioned are illustrated in this unit. The descriptive details of the employed data reported in Table 2 are performed by raw data (without the logarithmic specification). The Jarque-Bera test result is also extremely significant, showing that the data is not distributed normally. This will help us better understand how to choose the optimal econometric approach for non-normally distributed data.

**Table 2:** The following descriptive statistics is obtained on the raw data employed

Statistics	DIG	ENGHGs	ENT	FINR	GDP
*Mean*	46.65183	359148.3	1.660	35.81253	1.10E+12
*Median*	53.22051	63997.30	1.665	37.20833	3.03E+11
*Maximum*	99.01095	6302258.	4.590	49.04167	2.00E+13
*Minimum*	0.000000	1760.597	1.760	0.000000	9.14E+09
*Std. Dev.*	35.00683	951726.6	0.667	8.646370	2.57E+12
*Skewness*	-0.120313	5.145950	0.102	− 2.725126	5.008220
*Kurtosis*	1.421429	29.58412	4.489	11.65691	30.29987
*Jarque-Bera*	118.5651***	37787.69***	105.038***	4866.104***	39320.96***
*Prob*	0.000	0.000	0.000	0.000	0.000
*Sum Sq. Dev.*	1366408.	1.01E+15	315772.2	83357.09	7.35E+27
*Observations*	1116	1116	1116	1116	1116

The level of significance is determined by 1, 5, and 10% indicated through ***, ** and * respectively

The problem of a dependency across the cross-sectional units emerges frequently while handling panel data. The CD test may alter the parameters’ exact value and may originate from factors that were overlooked, which, if ignored, might lower the panel data’s quality (Phillips & Sul 2003). The CD test findings in A of Table 3 strongly support rejecting the null hypothesis of no cross-sectional dependency and accepting the alternative; the existence of cross-sectional dependence at a 1% level of significance. The outcomes of the SH test, as given in B of Table 3, indicate that the data model is affected by the heterogeneous slope. Considering the issue of CD and SH, we develop distinct econometric approaches that address the problem of CD and SH concurrently and effectively.

**Table 3:** Slope heterogeneity and cross-sectional dependence check

Variables	Statistics	Prob
*Panel A: CD test*		
*ENGHGs*	23.361***	0.000
*GDP*	130.763***	0.000
*FINR*	13.301***	0.000
*DIG*	133.113***	0.000
*ENT*	13.525***	0.000
*Panel B: SH Test*		
Model	Delta_tilde	Adjusted Delta_tilde
	40.613***	45.225***

The level of significance is determined by 1, 5, and 10% indicated through ***, ** and * respectively. Under the null hypothesis of cross-section independence, CD ~ N(0,1) *P*-values close to zero indicate data are correlated across panel groups

In order to avoid misleading regression’s outcomes, we analyze the stationarity properties of the variables under examination. The outcomes of Pesaran (2007) panel unit root test reported in Table 4 ratify the mixed order of integration, i.e., GDP is non-stationary at level, while ENGHGs and ENT are stationary at level only at 5% and 10% level of significance. Moreover, FINR and DIG are stationary at level *I*(0). However, all the variables under consideration became stationary at the first difference *I*(1). Hence, Table 5 confirms that the variables are of mixed order integration (*I*(0) and *I*(1)).

**Table 4:** Results of CIPS unit root

Variables	Level	First difference	Order of integration
	Trend and constant	Trend and constant	
*ENGHGs*	− 2.604*	− 5.563***	*I*(0)
*GDP*	− 2.218	− 4.115***	*I*(1)
*FINR*	− 3.058***	-	*I*(0)
*DIG*	− 4.069***	-	*I*(0)
*ENT*	− 1.888	− 5.228***	*I*(1)

The level of significance is determined by 1, 5, and 10% indicated

**Table 5:** Panel cointegration test

Statistics	Value	*Z*-statistics	*p* value
*G*^*t*^	− 6.303***	− 25.227	0.000
*G*^*a*^	− 20.606***	− 9.050	0.000
*P*^*t*^	− 36.908***	− 21.476	0.000
*P*^*a*^	− 24.230***	− 14.867	0.000

Asterisks denote a significance level of 10% (*), 5% (**) and 1% (***)

Given that the slope model is heterogeneous and there is cross-sectional dependency, this study employs the Westerlund (2007) test to test the cointegrating links among the variables. Based on the results of Westerlund (2007) reported in Table 5, the significant values (*G*_*t*_ and *G*_*a*_) (*P*_*a*_ and *P*_*t*_) confirm the cointegrating relationship among the groups and across the cross-sectional units. In terms of residual dynamics, this approach alleviates the common factor limitation on tests since the dynamics are naturally organized rather than residuals (Kremers et al. 1992).

The findings of the method of moment quantile regressions (MMQR) proposed by Machado and Silva (2019) reported in Table 6 provide several interesting findings. The outcomes of each variable, its interpretations and the rationale behind its association with ENGHGs emissions, are discussed in detail below.

**Table 6:** Method of moment quantile regression (MMQR)

Variables	Method of moment quantile regressions					
	Location	Scale	Quantiles			
			0.25th	0.50th	0.75th	0.90th
GDP	0.945***	− 0.042***	0.979***	0.947***	0.907***	0.878***
FINR	− 0.736***	− 0.067***	− 0.683***	− 0.733***	− 0.796***	− 0.842***
DIG	− 0.090***	0.002***	− 0.092***	− 0.090***	− 0.088***	− 0.086***
ENT	0.023**	− 0.058***	0.069***	0.026***	− 0.028**	− 0.068***

Asterisks denote a significance level of 10% (*), 5% (**), and 1% (***)

We found that GDP is significantly linked with ENGHGs emissions in all quantiles. A unit rise in GDP caused on average 0.90 units of ENGHGs emissions (thousand tons of CO_2_ equivalent). A rising income level is associated with improved living standards, which directly activates energy demands. This statement is also justified by the initial phase of environmental Kuznets curve; growth or rise in earnings is connected with environmental pollution. This study’s GDP-related findings are similar to that of Al-mulali and Sheau-Ting (2014), Al Mamun et al. (2014), Bekun et al. (2019), and Khan and Bin (2020). Hence, our proposed hypothesis I is confirmed based on the significantly positive association with ENGHG emissions in the selected OECD economies.

Next, we confirm that improvement in FINR index curbs the issue of ENGHGs emissions in selected high-income economies. Specifically, a unit improvement in the FINR index improves environmental quality by curbing on average of 0.73 units of ENGHGs emissions (thousand tons of CO_2_ equivalent) in all quantiles significantly. The FINR developed by ICRG assesses a country’s ability to finance its various administrative activities. A lower-financial-risk economy is recognized as more productive economy that can fulfill its liabilities. Furthermore, a country’s currency rate stability attracts foreign capital due to lower FINR (Hassan et al. 2021). This finding contradicts the prior studies of Hassan et al. (2021); less FINR economies contribute to the inflow of economic activity resulting in more emissions. The contradiction is due to that the study of Hassan et al. (2021) employed consumption-based carbon emissions as a dependent variable in their empirical model, which considers the emissions generated along the entire chain, excluding the emissions of exports and adding the emission of imports. Moreover, the FINR-related findings of this study are in line with the findings of Zhao et al. (2021); a unit rise in FINR can cut CO_2_ emissions by 0.002%, suggesting that FINR has an inhibitory effect on CO_2_ emissions. In this regard, hypothesis III is validated by the direct linkage of FINR and ENGHGs based on the findings of Table 6.

The impact of DIG on ENGHGs among all quantiles is negative; DIG is helpful to tackle ENGHGs. Precisely, a percent rise in DIG effectively restrains the ENGHG emissions on average of 0.090 (thousand tons of CO_2_ equivalent). Digitalization improves the effectiveness of public involvement in pollution management and makes environmental data more accessible and transparent (Sun and Hu 2021). It is logical that DIG promotes the speedy flow of scientific integration and usage of multiple factors, while minimizing energy consumption, improving social production efficiency, and reducing emissions (Yi et al. 2022). These findings are similar to that of Yi et al. (2022). However, the DIG-related findings of this study are inconsistent with the findings of Zhang et al. (2022), who conclude that digital economy intensifies carbon emissions in provincial level panel data of China.

**Fig. 6:**
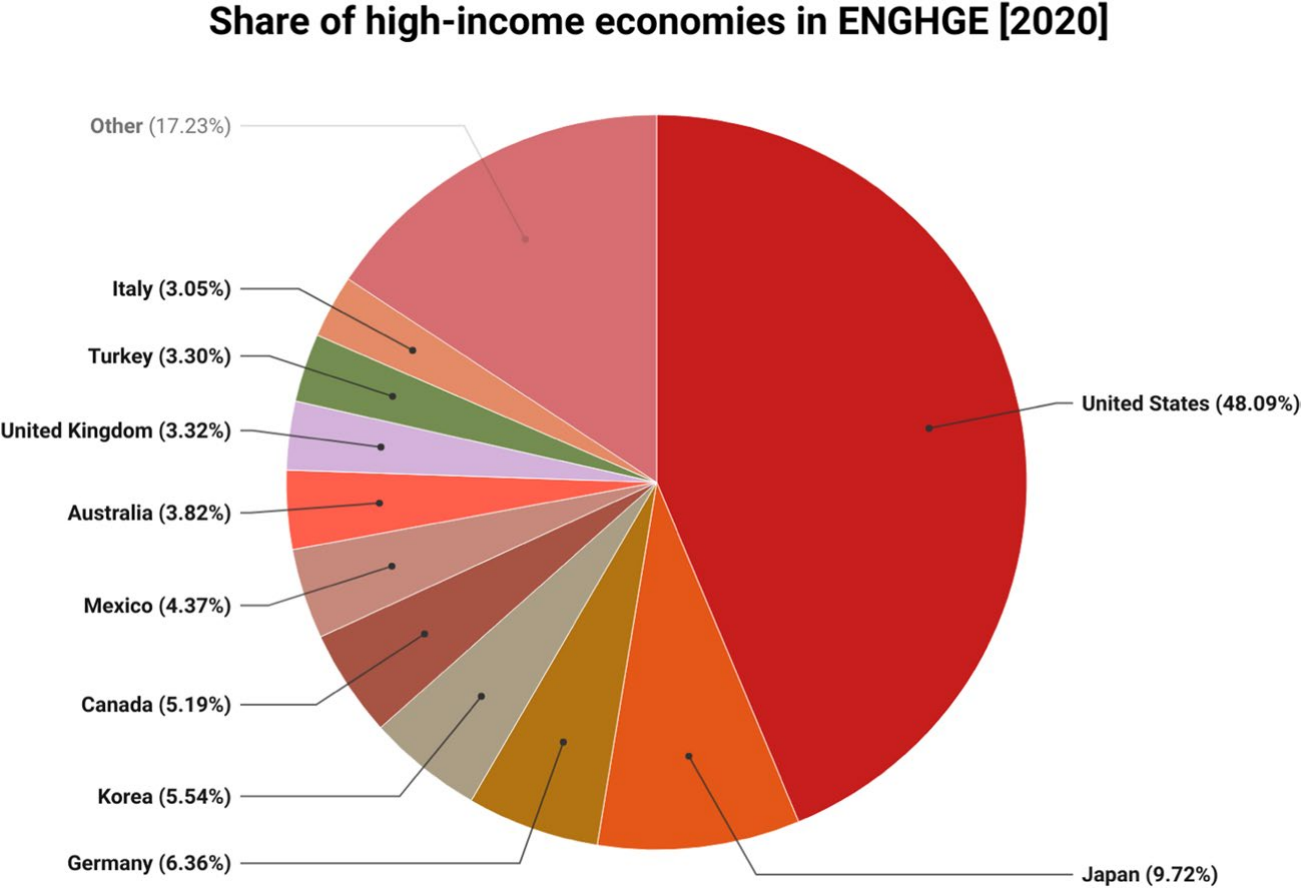
Energy-related tax (1990–2020)

**Fig. 7:**
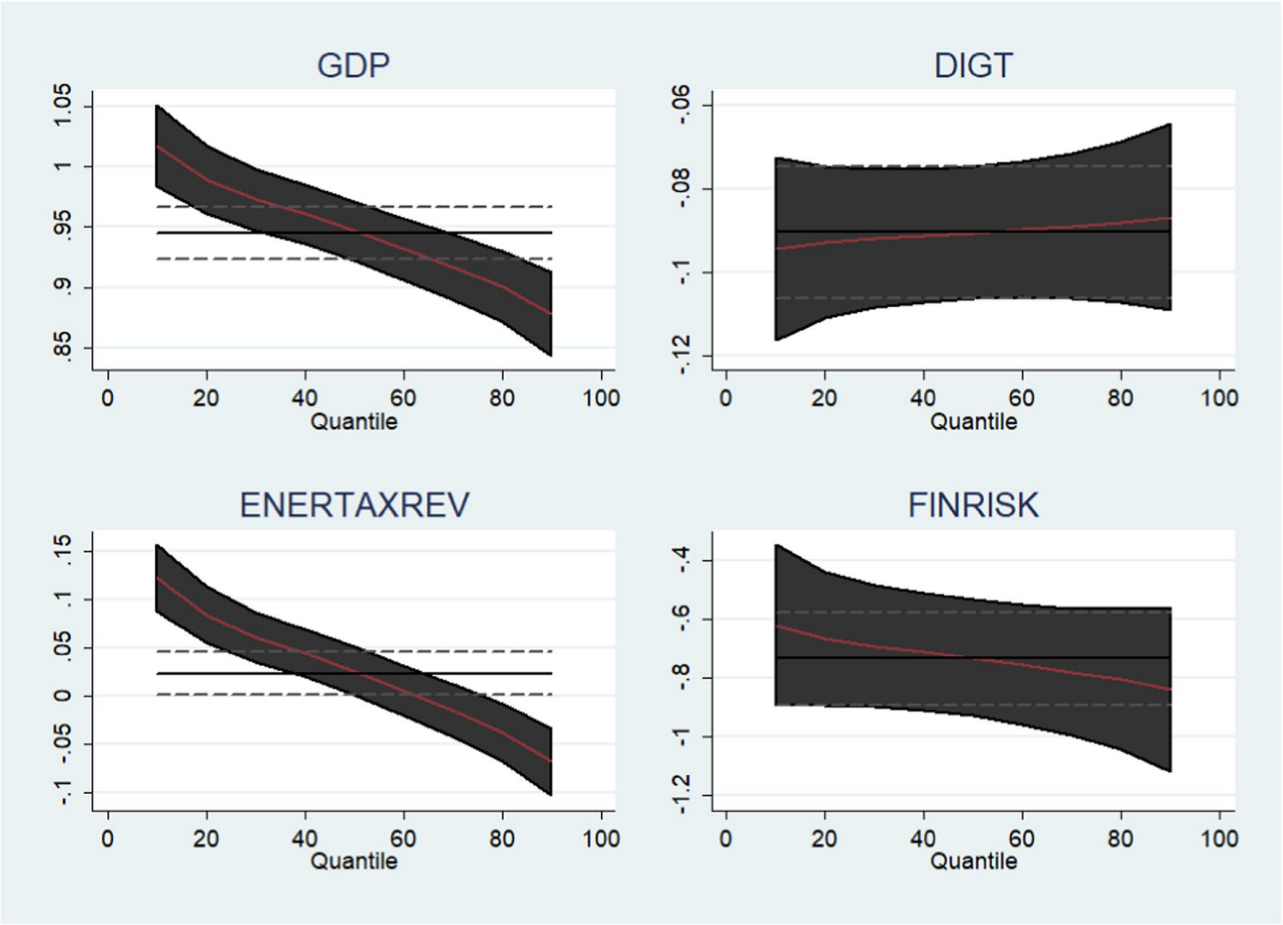
Visualization of fluctuations across quantiles (MMQR)

Interestingly, we confirmed that ENT has a heterogeneous effect on ENGHG emissions across various quantiles. To be specific, ENT is found to be the driver of ENGHGs in 25th and 50th quantiles significantly. However, in contrast, the study reveals that ENT significantly curbs ENGHGs in 75th and 90th quantiles. It is the rationale that initially, the energy tax is relatively modest in the first two quantiles. Nevertheless, in 75th and 90th quantiles, due to rise in ENT, it is discovered that certain high-income OECD economies might reduce ENGHG emissions with implementation of suitable ENT-related policies. The ENT-related findings of this study are consistent with the findings of Chien et al. (2021), who found that environmental tax significantly curbs PM2.5 and CO_2_ emissions in top Asian economies (Fig. 6). The visualiztion in Fig. 7 depict the impact fluctuation of each variable on ENGHGs in various quantiles.

### Robustness check (quantile regressions)

**Fig. 8:**
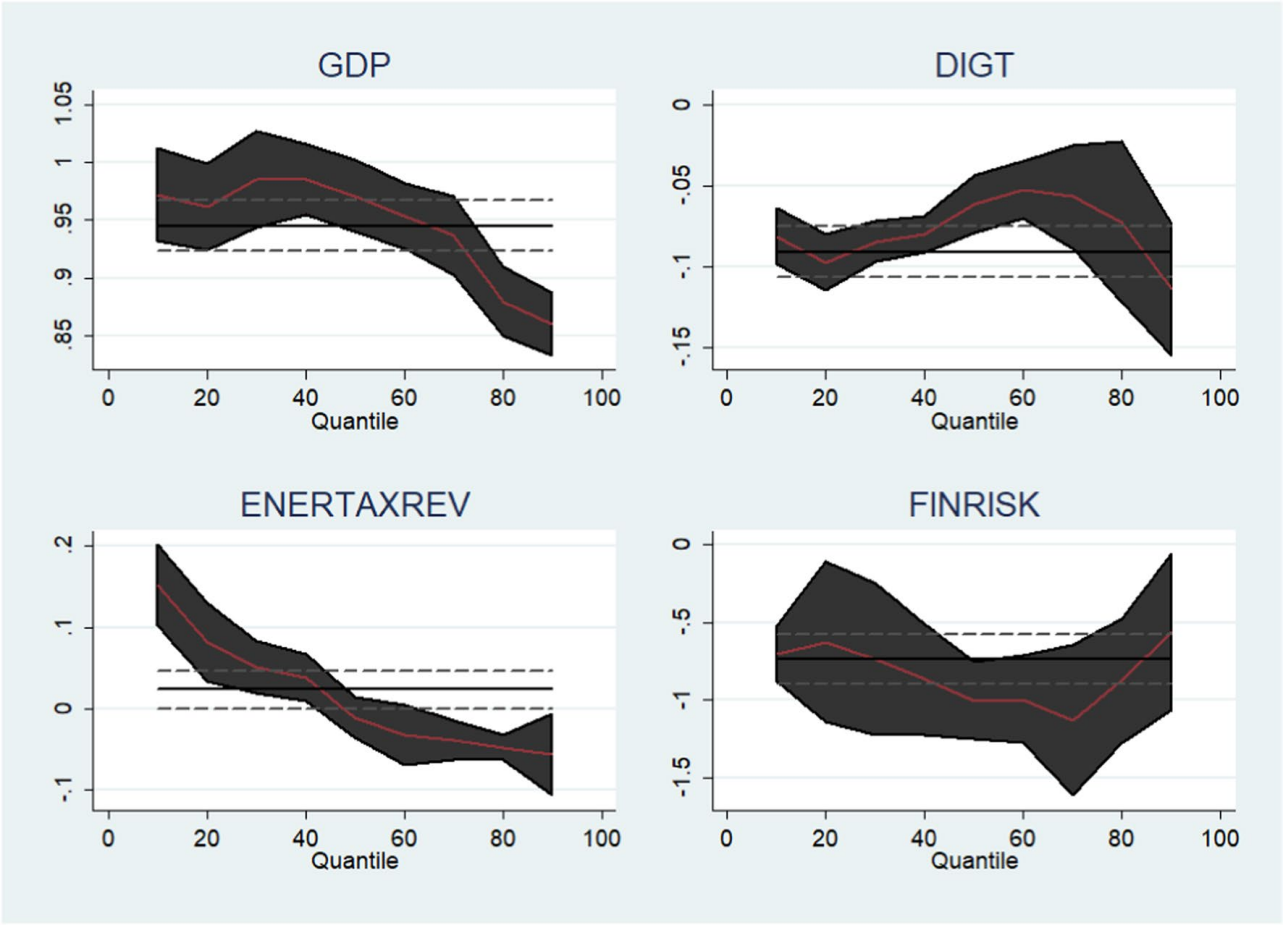
Visualization of fluctuations across quantiles (quantile regressions)

We next use bootstrap quantile regression to further confirm the empirical results of this investigation (Fig. 8). The MMQR results from Table 6 are confirmed by quantile regression, which is shown in Table 7. To be specific, it is further confirmed that GDP is activating ENGHGs in the selected OECD economies whereas improvement in FINR, ENT, and DIG sustains environmental quality by curbing ENGHGs in the region (Fig 8).

**Table 7:** Robustness check (quantile regression)

Variables	Quantile regressions			
	Quantiles			
	0.25	0.50	0.75	0.90
GDP	0.977***	0.971***	0.894***	0.860***
FINR	− 0.638**	− 1.004***	− 0.818***	− 0.564**
DIG	− 0.094***	− 0.061***	− 0.065***	− 0.113***
ENT	0.060***	− 0.010	− 0.052***	− 0.056**

Asterisks denote a significance level of 10% (*), 5% (**) and 1% (***)

The causal association between ENGHGs and employed predictors is verified by the Dumitrescu and Hurlin (2012) panel causality test. The outcomes in Table 8 indicate a two-way causal linkage between ENGHGs, FINR, GDP, DIG, and ENT significantly. Based on the bi-directional linkage, it is confirmed that any shock to ENGHGs will have significant implications for FINR, GDP, DIG, and ENT and the other way around.

**Table 8:**
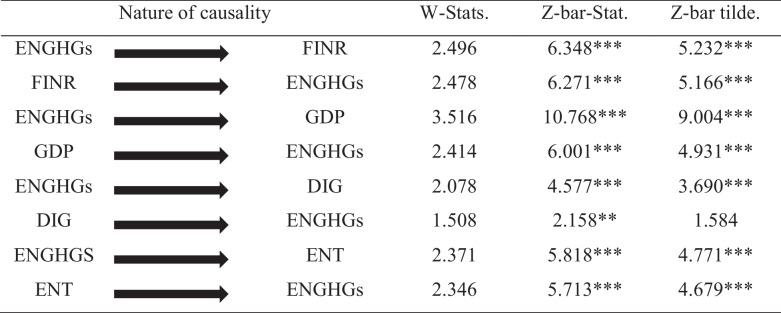
Pairwise Dumitrescu-Hurlin panel causality tests (2012)

Asterisks denote a significance level of 10% (*), 5% (**), and 1% (***)

## Conclusions and policy implications

Energy, a basic input to the economic system, poses significant challenges for sustaining environmental quality given the magnitude of change necessary to which many nations are locked into polluting and greenhouse gases emitted from energy sources. According to OECD (2017) report on green growth indicators, it is obvious that dependency on fossil fuel-based energy will continue to dominate the energy mix, and ENGHGs will double by 2050 if no immediate steps are taken.

The present study empirically examines any possible fluctuations arising in ENGHGs with any changes in GDP, FINR, DIG, and energy-related tax over the period of 1990–2020 in 36 high-income OECD economies. Based on the advanced econometric techniques, the present study provides several interesting findings; (1) the Jarque and Bera test in Table 3 suggests that the data is not normally distributed; (2) diagnostic tests reported in Table 4 confirm the presence of cross-sectional dependence and slope heterogeneity in the study model; (3) the Pesaran (2007) test mentioned in Table 5 validates the mixed order of variable integration I(0) and I(1); (4) the Westerlund (2007) panel cointegration test in 6 verifies the presence of cointegrating relationship among the group as well as in the whole panel significantly; (5) the novel method of moment quantile regression (MMQR) reported in Table 7 affirms rise in income level activates ENGHGs and improvement in FINR and DIG curbs ENGHGs significantly in all quantiles; (6) interestingly, ENT is found to be detrimental for environmental quality in 25th and 50th quantiles; however, it significantly limits ENGHGs in 75th and 90th quantiles; (7) the robustness check based on quantile regression in Table 8 further asserts the findings of MMQR; GDP causes surge, improvement in FINR and DIG curbs ENGHGs, whereas ENT has similar heterogeneous effect on ENGHGs (positive in 25th and 75th quantiles but negative in 75th and 90th quantiles); (8) finally, the panel causal check significantly validates a bi-directional linkage between ENGHGs, GDP, DIG, and ENT.

Taxing the most polluting energy sources is an effective way to cut emissions that threaten environmental quality. The revenue collected can be utilized to assist vulnerable families to make the low-carbon transition. Based on a new OECD study, 70% of energy-related emissions from industrialized and emerging economies remain untaxed, providing no motivation for the energy transition. The selected OECD nations must use a variety of policy tools to minimize fractions to net-zero energy transition in ways that are appropriate for their circumstances. Progressively raising emissions pricing and eliminating fossil fuel subsidies can assist governments in implementing more meaningful, realistic, and efficient climate policies. Such policy measures will be especially effective when coupled with strategies that promote developing low- and zero-carbon infrastructure and technology.

Based on the findings of this research, we conclude that although GDP is detrimental for sustaining environmental quality, improvement in FINR and DIG significantly tackles ENGHGs. Interestingly, it is found that due to the modest amount of ENT in the first two quantiles in selected high-income economies, ENT causes a surge in ENGHGs; however, due to the awareness of environmental pollution and climate change in the latter two quantiles, various developed nations have implemented strict environmental measures such as making it expensive to use fossil fuel–based energy sources.

The primary focus of the present study is to examine the empirical association between ENGHGs emissions and energy-related tax in 36 high-income OECD economies over the period of 1990–2020. The study is only based on a single empirical model for ENGHGs. However, future studies may also design model for energy-related carbon emissions and ENGHGs along with incorporating the same set of explanatory variables. This will provide comparatively interesting outcomes for both energy-related greenhouse gas emissions and energy-related carbon emissions.

## Data Availability

The dataset employed for empirical investigation in this article is available from the first author upon a reasonable request.
